# Modified Atmosphere Packaging Maintains the Sensory and Nutritional Qualities of Post-harvest Baby Mustard During Low-Temperature Storage

**DOI:** 10.3389/fnut.2021.730253

**Published:** 2021-09-06

**Authors:** Peixing Lin, Hongmei Di, Guiyuan Wang, Zhiqing Li, Huanxiu Li, Fen Zhang, Bo Sun

**Affiliations:** ^1^College of Horticulture, Sichuan Agricultural University, Chengdu, China; ^2^Institute of Pomology and Olericulture, Sichuan Agricultural University, Chengdu, China

**Keywords:** baby mustard, modified atmosphere packaging, sensory quality, antioxidant, glucosinolate, low-temperature storage

## Abstract

Baby mustard is a popular, yet highly perishable, *Brassica* vegetable. There is a need to develop effective methods for maintaining post-harvest qualities of baby mustard. Here, the lateral buds of baby mustard were packed in transparent polyethylene bags with no holes (M0), 6 mm in diameter holes (M1), or 12 mm in diameter holes (M2) and stored at 4°C. The effect of different modified atmosphere packaging (MAP) treatments on the sensory quality, health-promoting compounds, and antioxidant capacity was investigated by comparison with non-wrapped baby mustard. M1 and M2 delayed sensory quality deterioration and slowed declines in the content of ascorbic acid, total phenolics, and glucosinolates and antioxidant capacity during storage. M1 was most effective in prolonging the shelf life (three additional days compared with control lateral buds) and maintaining the content of glucosinolates. However, M0 accelerated the decline in the odor score, acceptability score, and ascorbic acid content and shortened the shelf life of baby mustard by more than 5 d compared with the control. These findings indicate that the effect of MAP treatment depends on the size of the holes in the bag. Based on these results, M1 was an alternative method for prolonging the shelf life and maintaining post-harvest qualities of baby mustard stored at 4°C.

## Introduction

Baby mustard (*Brassica juncea* var. *gemmifera*) is a variant of stem mustard that has become increasingly popular among consumers for its aesthetically pleasing appearance and high levels of health-promoting compounds such as glucosinolates, ascorbic acid, and phenolics (1–3). However, the lateral buds of baby mustard are perishable and susceptible to browning, dehydration, and the loss of health-promoting compounds during storage at ambient temperatures; indeed, maintaining post-harvest quality is one of the major challenges of the post-harvest processing of baby mustard (2, 3). There is thus a need to develop safe and effective methods for prolonging the shelf life of baby mustard and maintaining its sensory and nutritional qualities.

Our previous studies have shown that low temperature (4°C) storage can effectively maintain the sensory and nutritional quality of baby mustard (2). In addition, low temperature storage combined with modified atmosphere packaging (MAP) can delay senescence and reduce losses in quality during the storage of several vegetables, such as broccoli (4), lettuce (5), and fresh-cut watercress (6). MAP application can create an atmosphere of low O_2_ and high CO_2_, which can reduce the respiration rate of vegetables and delay senescence (7). MAP treatment can also maintain a high relative humidity (RH), which reduces water loss and helps maintain the visual quality of vegetables (4). In addition, MAP is simple and economical method, and can prevent cross-infection (5). These properties suggest that MAP could have commercial-scale applications. However, it has been reported that excessive accumulation of CO_2_ in MAP can damage the cell membrane and cause physiological injuries in mushroom (8). There is thus a need to determine the MAP treatment conditions suitable for specific produce during storage.

To the best of our knowledge, the effects of MAP on post-harvest quality of baby mustard have not been investigated. The aim of the current study was to evaluate the possibilities of MAP to maintain the sensory and nutritional qualities of post-harvest baby mustard and identify the optimal MAP treatment for baby mustard during post-harvest storage at 4°C. We hope that this work can provide an alternative application for the preservation of post-harvest baby mustard from the perspective of consumers and producers.

## Materials and Methods

### Plant Materials

Baby mustard (*Brassica juncea* var. *gemmifera* cv. Linjiang-Ercai), harvested early in the morning, was obtained from a local farm in Chengdu City, China, and transported to the laboratory within 2 h under ambient temperature. Fresh baby mustard with uniform size and absence of external damage was selected for experiments. Healthy lateral buds, the main edible parts of baby mustard, were removed and washed in an NaOCl solution (50 mg L^−1^) for 3 min, rinsed with tap water for 1 min, and then dried on blotting paper.

### MAP Treatment

The lateral buds were randomly assigned to four groups and stored in incubators at 4°C with a RH of 75% under continuous darkness. Approximately 300 g of baby mustard lateral buds was placed in three types of transparent polyethylene bags (18 cm × 25 cm, 80 μm thickness): (1) without holes (M0), (2) with 6 mm in diameter holes (eight holes, four holes on each side of the bag) (M1), and (3) with 12 mm in diameter holes (eight holes, four holes on each side of the bag) (M2). The O_2_ and CO_2_ transmission rates of polyethylene bags are 7.0 × 10^−7^ and 2.4 × 10^−6^ L m^−2^ s^−1^ at 25°C and standard pressure, respectively. As a control, lateral buds were stored without wrapping in transparent polypropylene containers without lids. Samples were taken after 0, 3, 6, 9, and 12 d. A bag of baby mustard lateral buds was collected as a repeat, and four repeats were used per sampling period. Several fresh samples were used for analyses of shelf life, sensory quality, and weight loss, and other samples were lyophilized in a freeze dryer and stored at −20°C for subsequent analyses of phytochemicals and antioxidant capacity.

### Quality Assessment

#### Shelf Life and Sensory Quality Evaluation

Shelf life and sensory quality of the baby mustard lateral buds were assessed daily and on sampling day, respectively. They were evaluated by a six-member panel, who are engaged in fresh produce research for at least 2 years. The samples were coded with random numbers to mask the treatment identity to minimize subjectivity and to ensure test accuracy. The lateral buds were considered to have reached the end of their shelf life when they became soft, shrank, and exhibited browning (3). Sensory attributes were quantified on a scale from 5 to 1 as follows. Color was rated using 5 = bright green without defects, 3 = lighter green with a few browning spots, and 1 = yellowish lateral buds with severe browning. Odor was rated using 5 = no off-odors, 3 = slight but obvious off-odor, and 1 = strong off-odor. Texture was rated using 5 = very tight and firm, 3 = slightly soften but acceptable, and 1 = very soften. Acceptance was rated using 5 = excellent and having a freshly harvested appearance, 3 = average, and 1 = unmarketable.

#### Weight Loss

Weight loss was analyzed as previously reported (3). Weight loss (%) was calculated by the formula (W_x_ – W_0_)/W_0_ × 100, where W_0_ is the weight at 0 d, and W_x_ is the weight at a certain day after storage.

#### Sucrose, Fructose, and Glucose Content

Sucrose, fructose, and glucose were extracted and analyzed as previously described (9). Freeze-dried samples (100 mg) were added to 5 mL of distilled water and homogenized for 1 min. The mixture was then extracted in a water bath at 80°C for 30 min. The supernatant was collected after centrifugation at 8,000 *g* at room temperature for 5 min, and filtered through 0.45 μm cellulose acetate filter, and then analyzed by high performance liquid chromatography (HPLC) using an Agilent 1260 instrument equipped with a refractive index detector (Agilent Technologies, Inc., Palo Alto, USA). Samples were separated at 35°C on an Agilent ZORBAX carbohydrate column (250 × 4.6 mm i.d.; 5 μm particle size) using 80% acetonitrile at a flow rate of 1.0 mL min^−1^. Content of sucrose, fructose, and glucose were determined using the standard curves for each sugar (Sangon Biotech Co., Ltd., shanghai, China). Results of sucrose, fructose, and glucose content were expressed as mg g^−1^ of dry weight.

#### Ascorbic Acid Content

Ascorbic acid content was determined according to the previous report (1). Fifty milligram of sample powder was extracted with 5 mL 1.0% oxalic acid, subsequently centrifuged 5 min at 4,000 *g*. Each sample was filtered through a 0.45 μm cellulose acetate filter. HPLC analysis of ascorbic acid was carried out using an Agilent 1260 instrument with a variable wavelength detector (VWD) detector (Agilent Technologies, Inc., Palo Alto, USA). Sample were separated on a Waters Spherisorb C18 column (150 × 4.6 mm i.d.; 5 μm particle size), using a solvent of 0.1% oxalic acid at a flow rate of 1.0 mL min^−1^. The amount of ascorbic acid was calculated from absorbance values at 243 nm, using authentic ascorbic acid (Sangon Biotech Co., Ltd., shanghai, China) as a standard. Result of ascorbic acid content was expressed as mg g^−1^ of dry weight.

#### Total Phenolics Content

Total phenolics were extracted with 10 mL of 50% ethanol and incubated at room temperature for 24 h in the dark. The suspension was centrifuged at 4,000 *g* for 5 min at room temperature. The supernatant was used for the measurements of total phenolics content and antioxidant activity. The supernatant was mixed with Folin-Ciocalteu reagent (Sangon Biotech Co., Ltd., shanghai, China), after 3 min, saturated sodium carbonate was added. The absorbance was measured at 760 nm with the spectrophotometer (Mapada Instruments Co., Ltd., Shanghai, China) as previously described (1). Gallic acid (Sangon Biotech Co., Ltd., shanghai, China) was used as a standard and the results were expressed as mg gallic acid equivalent g^−1^ dry weight.

#### Ferric Reducing Antioxidant Power (FRAP)

FRAP assay was performed according to the previous report (10). The extracted samples were added to the FRAP working solution incubated at 37°C and vortexed. The absorbance was then recorded at 593 nm using a spectrophotometer (Mapada Instruments Co., Ltd., Shanghai, China) after the mixture had been incubated in at 37°C for 10 min. FRAP values were calculated based on FeSO_4_·7H_2_O standard curves and expressed as μmol g^−1^ dry weight.

#### 2,2-Azinobis (3-Ethyl-Benzothiazoline-6-Sulfonic Acid) (ABTS) Assay

ABTS antioxidant activity was performed according to the previous report (1). An aliquot of 300 μL of each extracted sample was added to 3 mL of ABTS^+^ solution. The absorbance was measured spectrophotometrically (Mapada Instruments Co., Ltd., Shanghai, China) at 734 nm after exactly 2 h, and then the value was calculated.

#### Glucosinolate Composition and Content

Glucosinolates were extracted and analyzed as previously described (1). Freeze-dried samples (100 mg) were boiled in 5 mL water for 10 min. The supernatant was collected after centrifugation, and the residues were washed once with water, centrifuged, and then combined with the previous extract. The aqueous extract was applied to a DEAE-Sephadex A-25 column (Sigma Chemical Co., Saint Louis, USA). The glucosinolates were converted into their desulpho analogs by overnight treatment with 100 μL of 0.1% aryl sulphatase (Sigma Chemical Co., Saint Louis, USA), and the desulphoglucosinolates were eluted with 1 mL water. HPLC analysis of desulphoglucosinolates was carried out using an Agilent 1260 HPLC instrument equipped with a VWD detector (Agilent Technologies, Inc., Palo Alto, USA). Samples were separated at 30°C on a Waters Spherisorb C18 column (250 × 4.6 mm i.d.; 5 μm particle size) using acetonitrile and water at a flow rate of 1.0 mL min^−1^. Absorbance was detected at 226 nm. Glucosinolates were quantified by using *ortho*-Nitrophenyl β-D-galactopyranoside (Sigma Chemical Co., Saint Louis, USA) as the internal standard and considering the response factor of each glucosinolate. Result of glucosinolate content was expressed as μmol g^−1^ of dry weight.

### Statistical Analysis

To measure shelf life and visual quality, six replicates were prepared for each treatment. Other assays were performed in quadruplicate. Statistical analysis was performed using the SPSS package program version 18 (SPSS Inc., Chicago, IL, USA). Data were analyzed using one-way analysis of variance. Principal component analysis (PCA) was performed in SIMCA-P 11.5 Demo software (Umetrics, Sweden) with unit variance (UV)-scaling to decipher the relationships among samples (1). A time-related trajectory analysis based on two-dimensional PCA map was applied to visualize the temporal alterations of post-harvest quality changes under different MAP treatments (3).

## Results

### Sensory Analysis

**Figure 1:**
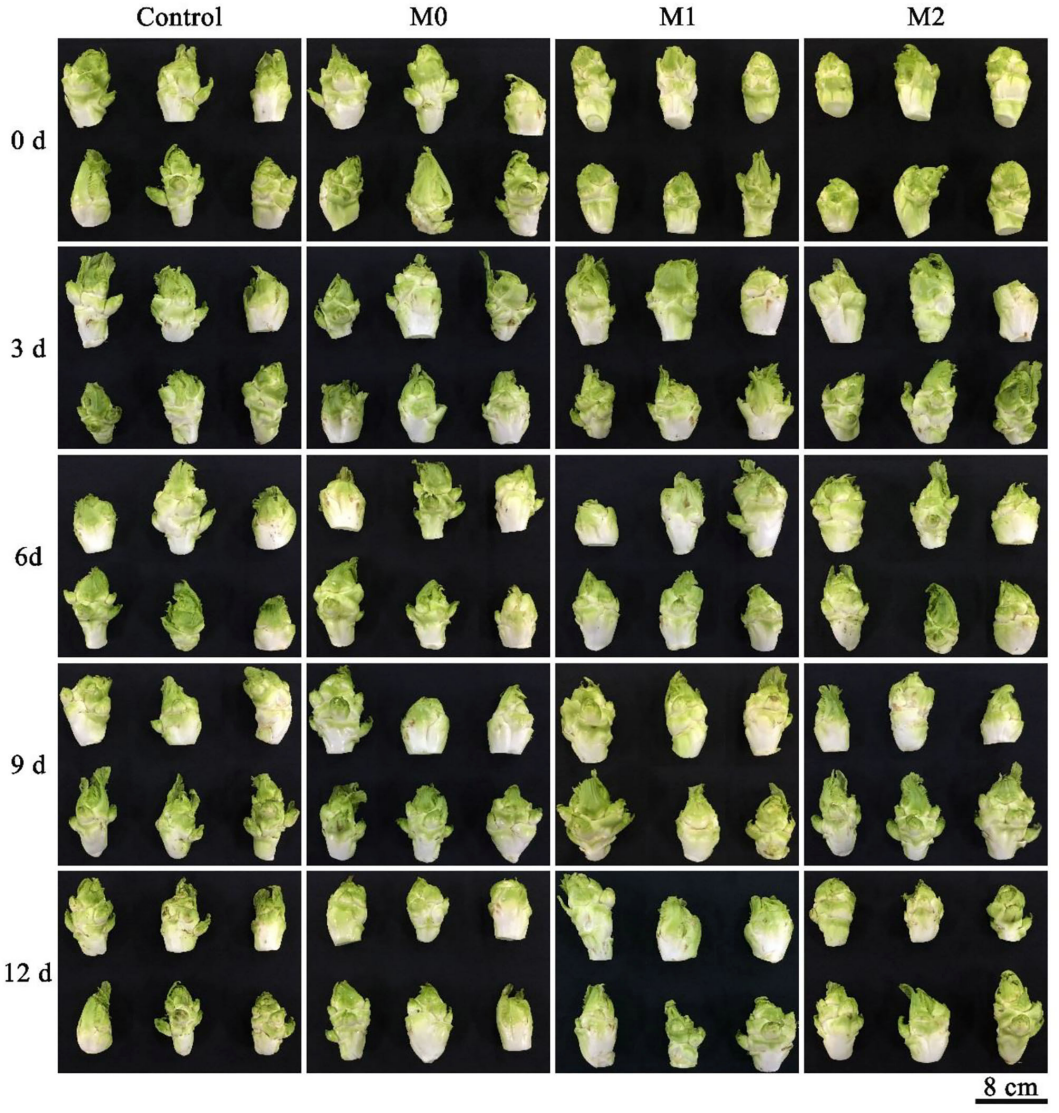
Appearance of different baby mustard lateral buds during storage at 4°C under different MAP treatments. M0 indicates packaging with no holes; M1 indicates packaging with 6 mm in diameter holes; and M2 indicates packaging with 12 mm in diameter holes. Scale bar = 8 cm.

**Figure 2:**
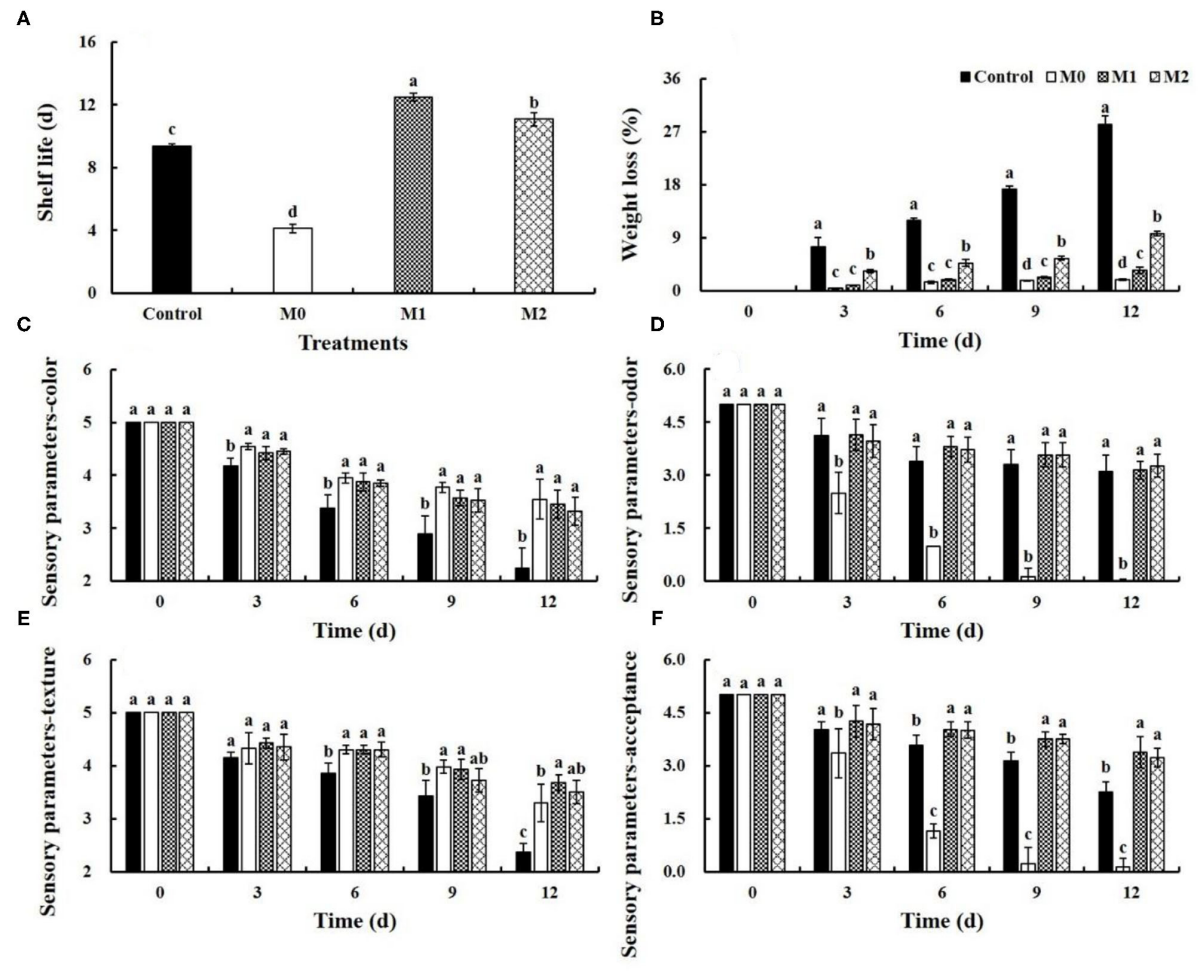
Shelf life **(A)**, weight loss **(B)**, and sensory parameters **(C–F)** of different baby mustard lateral buds during storage at 4°C under different MAP treatments. Sensory parameters include color, odor, texture, and acceptance of lateral buds. Different letters in **(A)** indicate statistically significant differences among treatments (*P* < 0.05), and different letters in **(B–F)** indicate statistically significant differences among treatments for each storage day (*P* < 0.05). M0 indicates packaging with no holes; M1 indicates packaging with 6 mm in diameter holes; M2 indicates packaging with 12 mm in diameter holes.

Baby mustard gradually deteriorated as lateral buds shriveled and browned on the peel during storage, and MAP treatments significantly delayed deterioration (Figure 1, Supplementary Figure 1). M1 and M2 significantly (*P* < 0.05) prolonged the shelf life of baby mustard after harvest (Figure 2A). The longest shelf life, which was observed in M1, was three additional days compared with the control. However, M0 shortened the shelf life of baby mustard after harvest by more than 5 d compared with the control.

Weight loss is one of the key sensory characteristics for evaluating fresh vegetable quality. Weight loss increased during storage time. Weight loss in the control was the most dramatic, which decreased by 28.3% at 6 d. MAP treatments significantly (*P* < 0.05) suppressed weight loss. The weight loss in all MAP treatments was <10% at 12 d (Figure 2B).

Throughout the entire storage period, the sensory parameter scores of both MAP-treated and control baby mustard decreased gradually. Compared with the control, higher color and texture scores of lateral buds were observed under MAP treatments, and no significant differences were observed between the different MAP treatments (Figures 2C,E). The odor score of M0 decreased rapidly during storage, and the odor score of the control, M1, and M2 slightly decreased during storage (Figure 2D). Compared with the control, M0 accelerated the decrease in acceptance scores; however, M1 and M2 delayed the decline in acceptance scores (Figure 2F).

### Soluble Sugars

**Figure 3:**
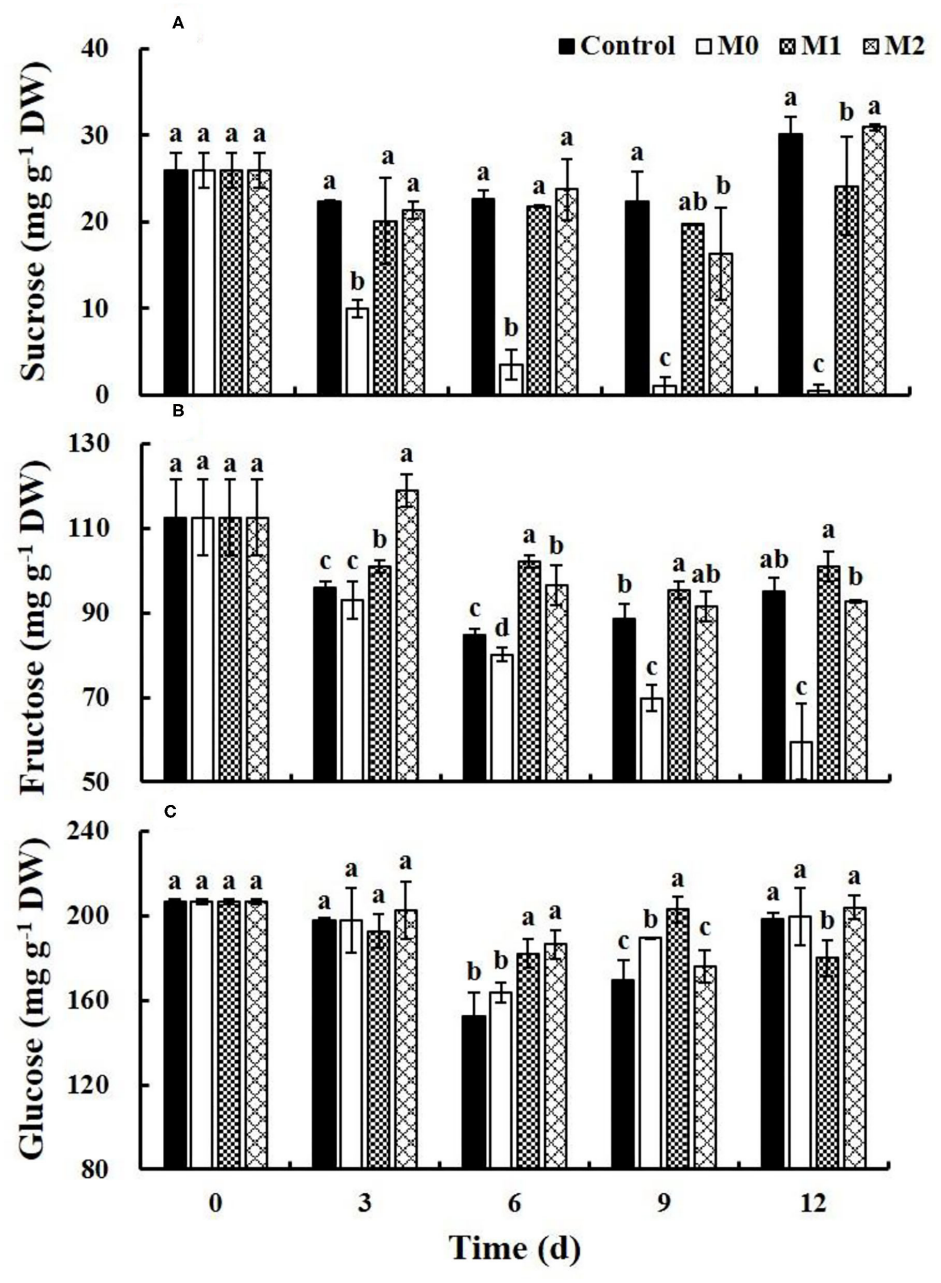
Content of sucrose **(A)**, fructose **(B)**, and glucose **(C)** of different baby mustard lateral buds during storage at 4°C under different MAP treatments. Different letters in the figure indicate statistically significant differences among treatments for each storage day (*P* < 0.05). M0 indicates packaging with no holes; M1 indicates packaging with 6 mm in diameter holes; M2 indicates packaging with 12 mm in diameter holes.

Sucrose, fructose, and glucose were identified in baby mustard, and glucose was the most abundant (Figure 3). The content of sucrose slightly decreased early in the storage period and increased later in the storage period in the control, M1, and M2. However, the increase in sucrose in M1 was significantly (*P* < 0.05) slower than the increase in the control in the late storage period, and the content of sucrose was 19.9% lower in M1 than in the control at the end of storage. The content of sucrose gradually decreased in M0 and was significantly (*P* < 0.05) lower in M0 than in the other treatments throughout storage (Figure 3A). The fructose content decreased during storage in the MAP treatments and the control, and the change in the fructose content was the smallest in M1 (Figure 3B). The glucose content first decreased and then increased during storage, and the change in M1 was the smallest (Figure 3C).

### Ascorbic Acid and Total Phenolics

**Figure 4:**
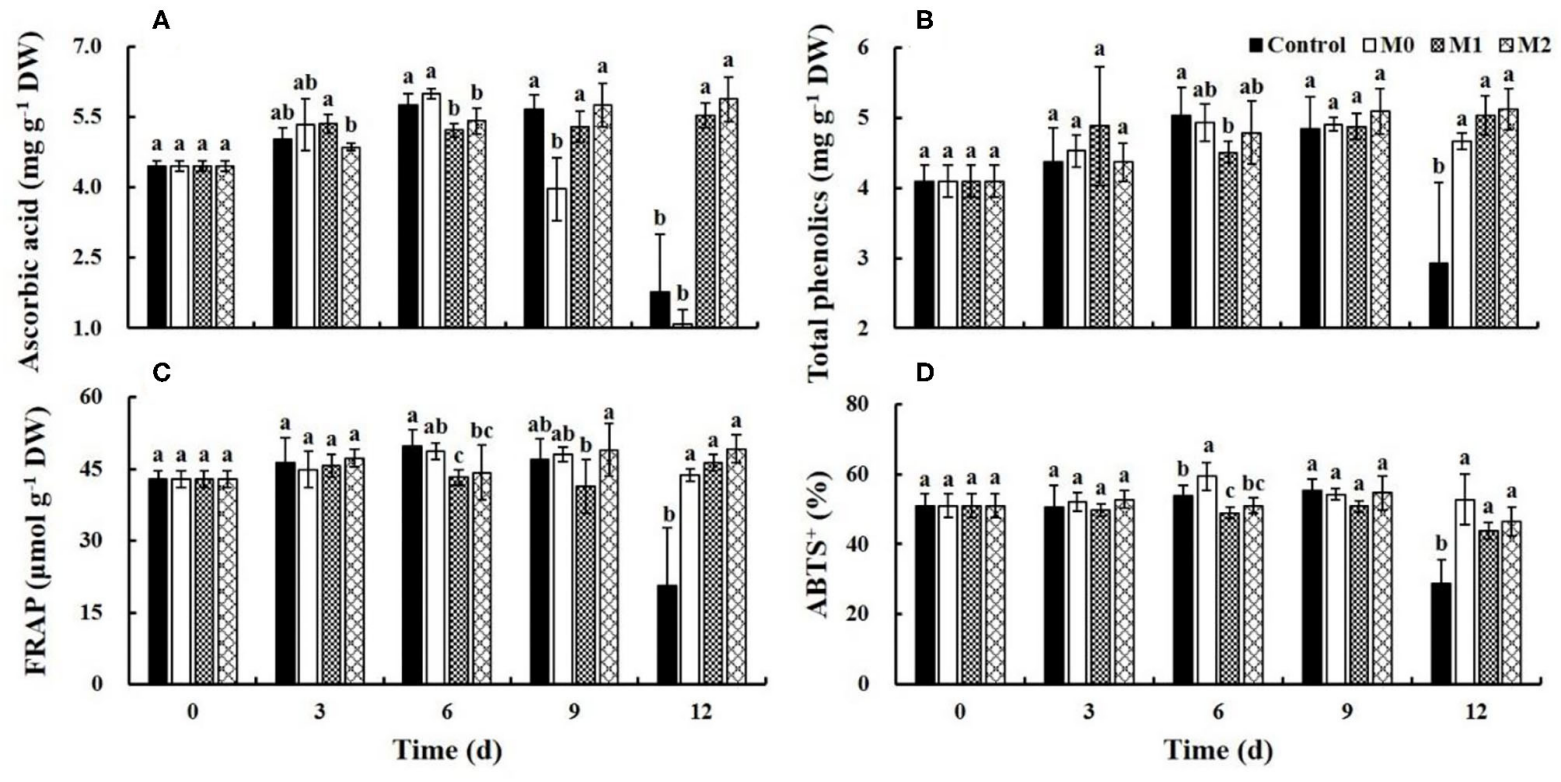
The main antioxidants content and antioxidant capacity level of different baby mustard lateral buds during storage at 4°C under different MAP treatments. **(A)** Ascorbic acid content; **(B)** total phenolics content; (C) FRAP level; **(D)** ABTS^+^ level. Different letters in the figure indicate statistically significant differences among treatments for each storage day (*P* < 0.05). M0 indicates packaging with no holes; M1 indicates packaging with 6 mm in diameter holes; M2 indicates packaging with 12 mm in diameter holes.

The ascorbic acid content in the control and M0 gradually increased during the first 6 d of storage but decreased sharply after 9 and 6 d, respectively; at the end of storage, the ascorbic acid content in the control and M0 decreased by 68.6 and 81.8%, respectively. The ascorbic acid content in M1 and M2 treatments was significantly (*P* < 0.05) higher than that in control and M0 treatment at the end of storage. The ascorbic acid content in M1 and M2 increased by 24.1 and 31.7%, respectively, over the entire storage period (Figure 4A). The total phenolics content in the control increased early during storage and decreased rapidly after 9 d of storage by 39.5%. The total phenolics content increased in M0, M1, and M2 during storage by 14.0, 23.1, and 25.2%, respectively, over the entire storage period. However, there is no significant (*P* < 0.05) difference between MAP treatments (Figure 4B). Overall, MAP treatments, especially M1 and M2, promoted the accumulation of ascorbic acid and phenolics in baby mustard.

### Antioxidant Capacity

The FRAP level in the control increased in the early stage of storage and decreased rapidly after 9 d of storage by 56.2%; however, in M0, M1, and M2, the FRAP level increased by 1.9, 7.9, and 14.8%, respectively, over the entire storage period (Figure 4C). The ABTS levels in the control and MAP treatments were relatively stable in the first 9 d of storage. After 9 d of storage, the ABTS levels in the control decreased significantly (down by 47.7%), and that in M1 and M2 decreased slightly, and there were no significant differences between MAP treatments (Figure 4D). In short, MAP treatment was beneficial for maintaining the antioxidant capacity, and antioxidant capacity decreased in the control after 9 d of storage.

### Glucosinolates

**Figure 5:**
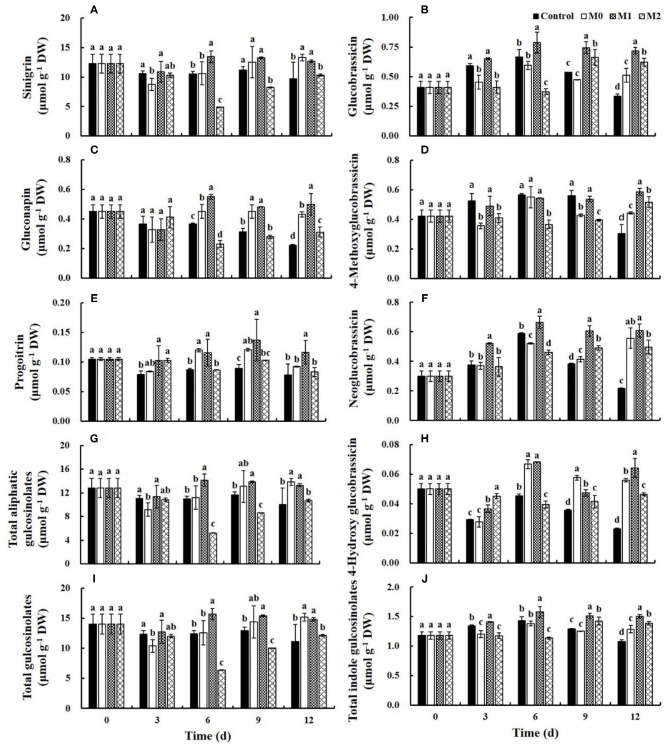
Glucosinolates content of different baby mustard lateral buds during storage at 4°C under different MAP treatments. **(A)** sinigrin; **(B)** glucobrassicin; **(C)** gluconapin; **(D)** 4-methoxyglucobrassicin; **(E)** progoitrin; **(F)** neoglucobrassicin; **(G)** total aliphatic gulcosinolates; **(H)** 4-hydroxy glucobrassicin; **(I)** total gulcosinolates; **(J)** total indole gulcosinolates. Different letters in the figure indicate statistically significant differences among treatments for each storage day (*P* < 0.05). M0 indicates packaging with no holes; M1 indicates packaging with 6 mm in diameter holes; M2 indicates packaging with 12 mm in diameter holes.

Three aliphatic and four indole glucosinolates were identified by HPLC in the lateral buds of baby mustard (Figure 5). The content of aliphatic glucosinolates in the control slightly decreased during the first 3 d of storage and remained basically unchanged thereafter, with the exception of gluconapin. The content of aliphatic glucosinolates in M1 increased after 3 d of storage and was significantly higher in M1 than in the control at the end of the storage period; the content of sinigrin, gluconapin, progoitrin, and total aliphatic glucosinolates was 1.3-, 2.2-, 1.5-, and 1.3-fold higher in M1 than in the control, respectively (Figures 5A,C,E,G). The indole glucosinolate content in the control increased during the first 6 d of storage and then decreased, with the exception of 4-hydroxyglucobrassicin. The indole glucosinolate content in M1 also increased during the first 6 d of storage but remained relatively unchanged thereafter. At the end of storage, the content of glucobrassicin, 4-methoxyglucobrassicin, neoglucobrassicin, 4-hydroxyglucobrassicin, and total indole glucosinolates was 2.1-, 1.9-, 2.7-, 2.8-, and 1.4-fold higher in M1 than in the control, respectively (Figures 5B,D,F,H,J). Because of the large proportion of sinigrin, the change in the content of total glucosinolates was similar to the change in sinigrin during storage (Figure 5I). Furthermore, the most of glucosinolates content in M0 and M2 treatments was also significantly (*P* < 0.05) higher than that in the control, but significantly (*P* < 0.05) lower than that in M1 treatment. These results indicated that MAP treatment could effectively preserve the content of glucosinolates, and M1 was the most effective.

### Time-Related Trajectory Analysis

**Figure 6:**
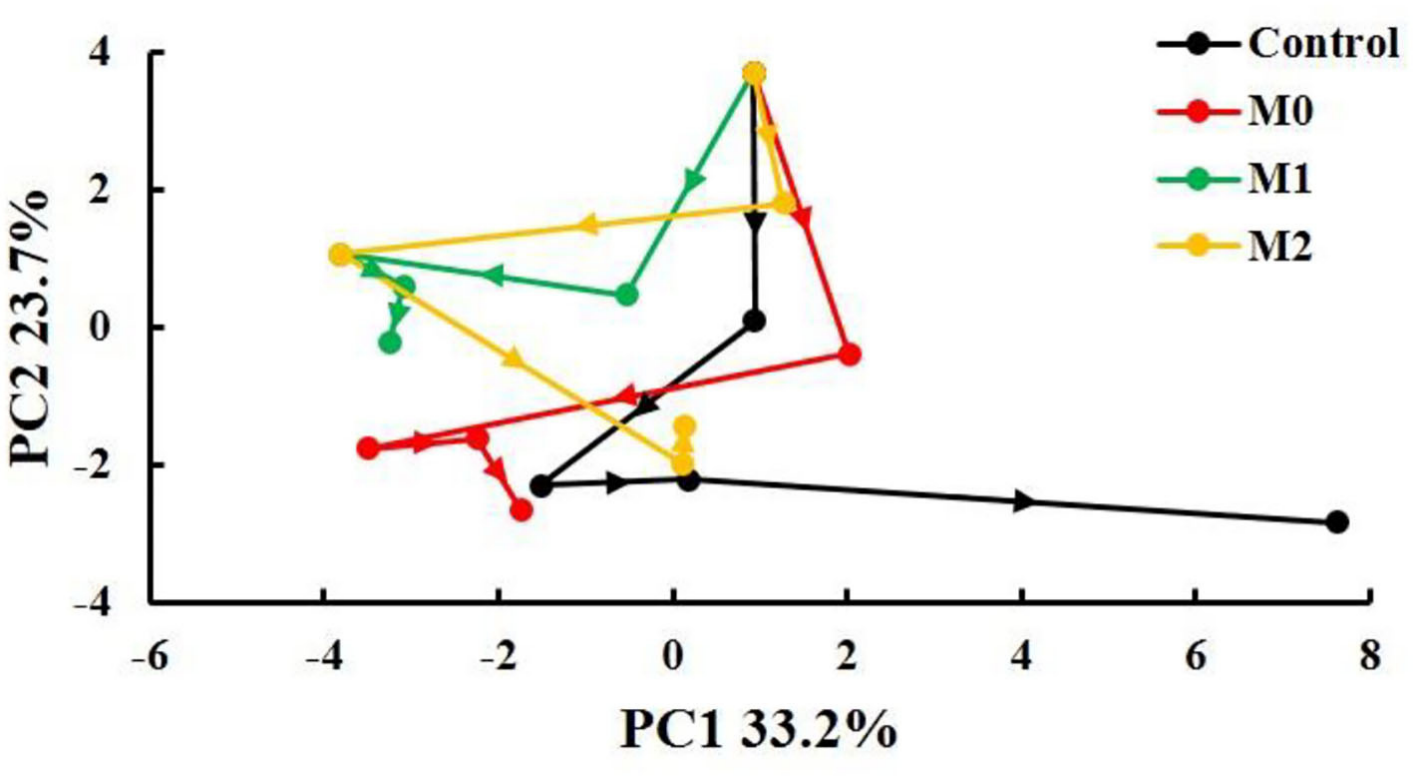
Time-related trajectory plot showing the dynamic time-related responses of sensory and nutritional qualities of different baby mustard lateral buds during storage at 4°C under different MAP treatments. M0 indicates packaging with no holes; M1 indicates packaging with 6 mm in diameter holes; M2 indicates packaging with 12 mm in diameter holes.

The points representing different storage times and treatments were notably separated, and longer distances between points correspond to greater deterioration in quality over a certain storage period. The quality of baby mustard in the control decreased rapidly and continuously during the entire storage period; that in M0 and M1 decreased mainly in the first 6 d of storage; and that in M2 decreased mainly in the first 9 d of storage. In general, the total length of the fold line was shortest in M1, which indicated that the deterioration in the quality of baby mustard in M1 was the lowest (Figure 6).

## Discussion

### Sensory Quality

In this study, M1 and M2 extended the shelf life and delayed weight loss and declines in sensory parameter scores in baby mustard during post-harvest storage compared with the control (Figures 1, 2). The greater visual quality under M1 and M2 likely results from the delay in the senescence of fresh produce associated with decreased respiration rates (5, 11). A study of lettuce indicated that fresh lettuce consumes O_2_ and produces CO_2_ when packed, which reduces its respiratory rate, extends its shelf life, and maintains its visual quality (5). Similar findings have also been obtained for broccoli florets (4), *Toona sinensis* (12), and watercress (6).

The weight loss of MAP treated baby mustard was reduced (Figure 2B), which may be due to the ability of the plastic film to restrict the diffusion of water vapor, which increases the water vapor pressure and relative humidity inside the package (4, 12). A shriveled appearance is associated with weight loss (5). Thus, the difference in weight loss between the control and the MAP-treated baby mustard may partially explains why the appearance of the MAP-treated baby mustard was superior to that of the control.

M0 shortened the shelf life and promoted declines in odor and acceptance scores in baby mustard during storage compared with the control (Figure 2). This may stem from the fact that M0 increased the production of acetaldehyde and ethanol by anaerobic metabolism because of respiratory consumption in a tightly sealed environment, producing an off-odor (4). Similar observations have also been made in broccoli florets (13). Thus, the post-harvest preservation effect of MAP may depend on the size of the punched hole in the film (4).

### Soluble Sugars

Sugars are an essential source of energy for extending the post-harvest life of perishable horticultural commodities (14). MAP treatment can create a low O_2_ and high CO_2_ atmosphere. Along with the decrease of O_2_ concentration and the increase of CO_2_ concentration when packed, the aerobic respiration of vegetables will decrease (7). However, when the O_2_ concentration is too low in the bag, baby mustard may produce anaerobic respiration (5). Anaerobic respiration provides less energy than aerobic respiration, and consumes more respiratory substrates in life activities, which accelerates the senescence of vegetables (15). The content of sucrose and fructose decreased rapidly during M0 (Figures 3A,B). This may be explained by the too low oxygen concentration caused by respiratory consumption in a tightly sealed environment, resulting in anaerobic respiration, thereby accelerating sugar consumption (15). The content of fructose and glucose in M1 changed less compared with other treatments (Figures 3B,C). This may due that the aerobic respiration of baby mustard is inhibited in M1 treatments (7). Meanwhile, the existence of holes ensures that there is a certain gas exchange between the microenvironment in the bag and the external environment to avoid the occurrence of anaerobic respiration. Therefore, compared with other treatments, M1 is most conducive to reducing respiration, which was beneficial to inhibit fructose and glucose degradation.

Sucrose content can be increased by environmental stress during post-harvest storage (16), and this is supported by our previous studies (2, 3). In this study, the increase in the sucrose content was slower in M1 than in the control in the late storage period (Figure 3A). This may be explained by the reduction in the respiration rate in M1 during post-harvest storage, which reduced environmental pressure.

Similar to the control, the glucose content in all treatments increased in the late storage (Figure 3C). A study of broccoli also found that the glucose content increased in the late storage (17). However, the reason for this pattern remains unclear, and additional studies are needed to clarify the underlying mechanism.

### Antioxidants and Antioxidant Activity

Ascorbic acid is often used as an indicator of nutrient quality in vegetables because of its lability (18). Previous studies have shown that ascorbic acid can be biosynthesized during storage to regenerate tocopherols to prevent the deterioration of plant tissues (18) and can effectively reduce the content of reactive oxygen species to inhibit tissue browning (12, 19, 20). The ascorbic acid content in baby mustard increased in the early stage of storage. However, in the late storage period, the ascorbic acid content decreased rapidly in the control and M0 but remained stable in M1 and M2 (Figure 4A). Similar results also found on *Toona sinensis*, which ascorbic acid content increased in the early stage of storage, however, when the storage prolonged, the ascorbic acid content declined with advancement in senescence (12). The decrease in ascorbic acid content in the control and M0 treated baby mustard during the late storage period may be due to the accelerated degradation of ascorbic acid, resulting in the degradation and utilization rates of ascorbic acid were likely higher than its biosynthesis rate. While, it is possible that the degradation and utilization rate of ascorbic acid and its biosynthesis rate may remain relatively balanced under M1 and M2 treatments in the late stage of storage. In addition, water loss can also hasten ascorbic acid degradation (21), and MAP significantly slowed weight loss and thus the decrease in ascorbic acid content.

Vegetables produce more secondary metabolites including phenolics to defend against stress during post-harvest storage (22). A similar situation was observed in this study: the total phenolics content of baby mustard increased early during post-harvest storage. However, the total phenolics content in the control decreased after 9 d of storage, which may stem from the acceleration of total phenolics degradation, as the degradation and utilization rates of phenolics were likely higher than the rate of biosynthesis of phenolic compounds late in the storage period (2, 22). However, the total phenolics content in MAP treatments remained relatively stable in the late storage. A previous study of medlar fruit showed that the loss of phenols during the storage can be reduced with MAP (23). An appropriate gas composition also delays the loss of phenols during the storage of guava fruit (24).

The antioxidant activity of fresh vegetables is largely related to the amount of phenolics (7, 25). The observed antioxidant activities in this study are consistent with this previous observation.

### Glucosinolates

Glucosinolates not only contribute to taste and flavor but also show anticancer activity by inhibiting the growth of tumor cells (2, 26, 27). However, baby mustard face a series of stresses after harvesting and during storage that may trigger the metabolism of glucosinolates and alter glucosinolate levels (28). The content of aliphatic glucosinolates in baby mustard in all treatments was decreased early during storage. This may stem in part to the contact of myrosinase and aliphatic glucosinolates in the pre-storage pre-treatment and the subsequent hydrolysis of glucosinolates. After storage for 3 d, the content of most aliphatic glucosinolates in the control remained relatively stable. This may stem from the fact that low temperature (4°C) storage retards the decline in the content of glucosinolates in baby mustard during post-harvest storage (2). Although the glucosinolates content decreased as the quality of baby mustard deteriorated during storage, the content of aliphatic glucosinolates in M1 increased after 3 d of storage. This finding is consistent with previous studies of broccoli showing that the content of aliphatic glucosinolates, including glucoiberin and glucoraphanin, in broccoli increased under controlled atmosphere treatments (28–30). MAP treatment significantly delayed the weight loss of baby mustard, indicating that cell integrity was enhanced, and cell damage was lower, under MAP treatment. Consequently, the bursting of vacuoles and the contact between glucosinolates and myrosinase were delayed under MAP treatment during storage (2). Inactivation of myrosinase by elevated CO_2_ concentrations (28, 31) might also explain the decreased degradation of glucosinolates observed in this study. In addition, the biosynthesis of aliphatic glucosinolates might be induced during controlled atmosphere storage (28).

Previous studies have shown that stress induces the biosynthesis of indole glucosinolates (32, 33). Similar observations were made in this study: the indole glucosinolates of baby mustard increased early during post-harvest storage. However, similar to aliphatic glucosinolates, the content of indole glucosinolates in the control decreased as the quality of baby mustard decreased during storage. The gas composition surrounding baby mustard gradually became suitable within the package, and this might explain the increase in the indole glucosinolates content in the MAP treatments.

## Conclusion

The visual quality and health-promoting compounds in the lateral buds of baby mustard were more effectively maintained in M1 and M2 than in the unwrapped control. M1 was the most effective for extending the shelf life and maintaining the content of glucosinolates. However, baby mustard in M0 developed a foul aroma or off-flavors during storage, which led to a decrease in the odor and acceptance score and shortened the shelf life compared with the control. Overall, this study suggests that M1 is a simple, economical, and effective method for maintaining the sensory quality and health-promoting compounds of baby mustard at low temperature (4°C). In addition, microbial analysis has been carried out in several similar studies. Although the problem of microbial contamination in post-harvest baby mustard is not serious, considering the importance of food safety, microbial analysis of baby mustard during the storage should also be done in future research.

## Data Availability Statement

The original contributions presented in the study are included in the article/Supplementary Material, further inquiries can be directed to the corresponding author/s.

## Author Contributions

HL and BS designed the experiments. PL, HD, and GW conducted the experiments. PL, FZ, and ZL analyzed the data. PL and HD wrote the manuscript. FZ and BS revised the manuscript. FZ and HL provided the financial support. All authors have read and agreed to the published version of the manuscript.

## Conflict of Interest

The authors declare that the research was conducted in the absence of any commercial or financial relationships that could be construed as a potential conflict of interest.

## Publisher's Note

All claims expressed in this article are solely those of the authors and do not necessarily represent those of their affiliated organizations, or those of the publisher, the editors and the reviewers. Any product that may be evaluated in this article, or claim that may be made by its manufacturer, is not guaranteed or endorsed by the publisher.

## Supplementary Material

Supplementary Figure 1 Appearance of different baby mustard lateral buds with bags during storage at 4°C under different MAP treatments. M0 indicates packaging with no holes; M1 indicates packaging with 6 mm in diameter holes; M2 indicates packaging with 12 mm in diameter holes.

Supplementary Figure 2 High performance liquid chromatography chromatograms. **(A)** Soluble sugars; **(B)** Ascorbic acid; **(C)** Glucosinolates. 1, 2, 3, 4, 5, 6, 7, and 8 indicate progoitrin, sinigrin, gluconapin, 4-hydroxy glucobrassicin, ortho-Nitrophenyl β-D-galactopyranoside, glucobrassicin, 4-methoxyglucobrassicin, and neoglucobrassicin, respectively.

Supplementary Table 1 Sensory and nutritional qualities in the lateral buds of baby mustard during storage.
