# Supplementary figures and images for: Modified Atmosphere Packaging Maintains the Sensory and Nutritional Qualities of Post-harvest Baby Mustard During Low-Temperature Storage
Source: Front Nutr. 2021 Sep 6;8:730253. doi: 10.3389/fnut.2021.730253 (PMC8450372; doi:10.3389/fnut.2021.730253)

Supplementary Material

## Supplementary Figure 1


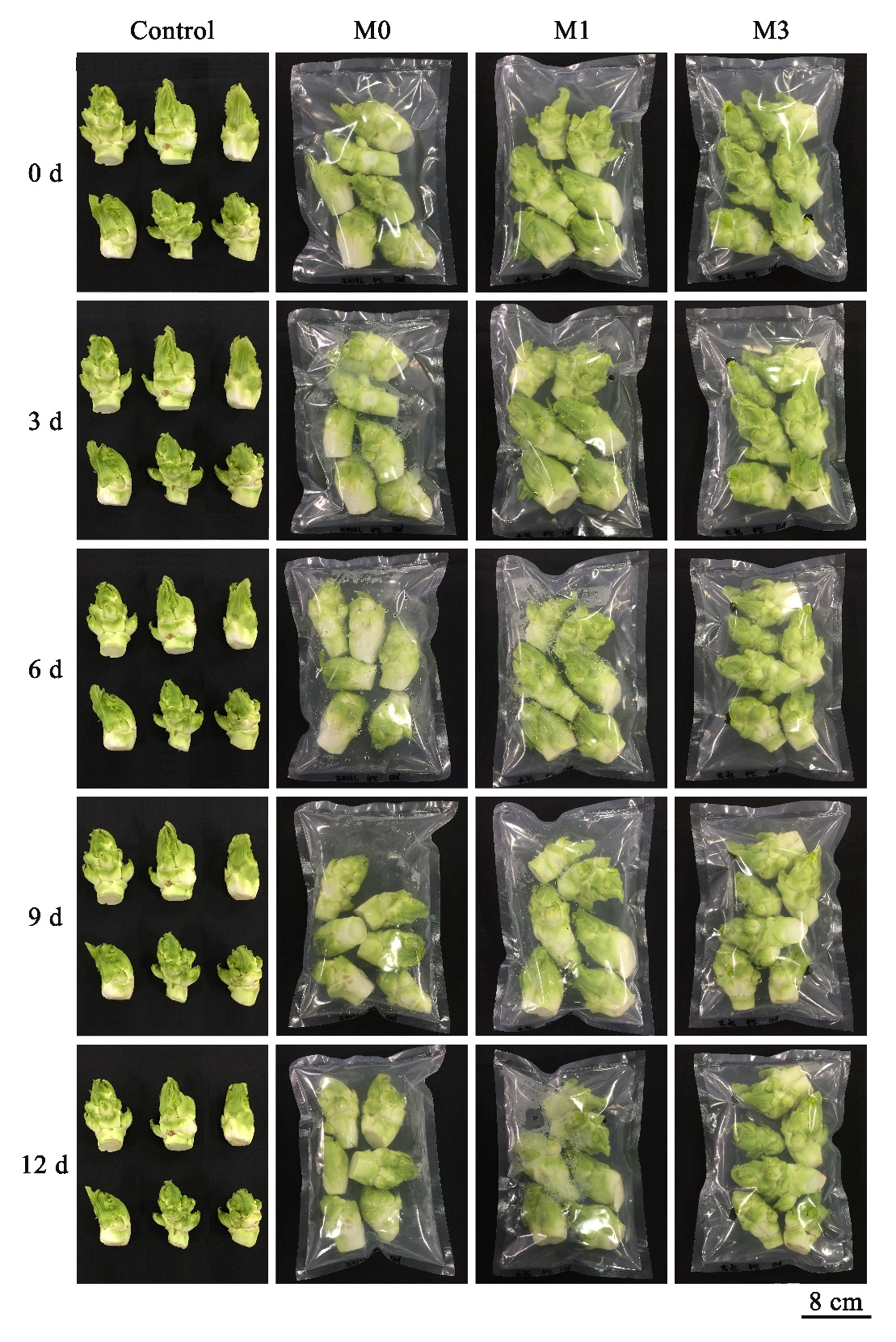


## Supplementary Figure 2


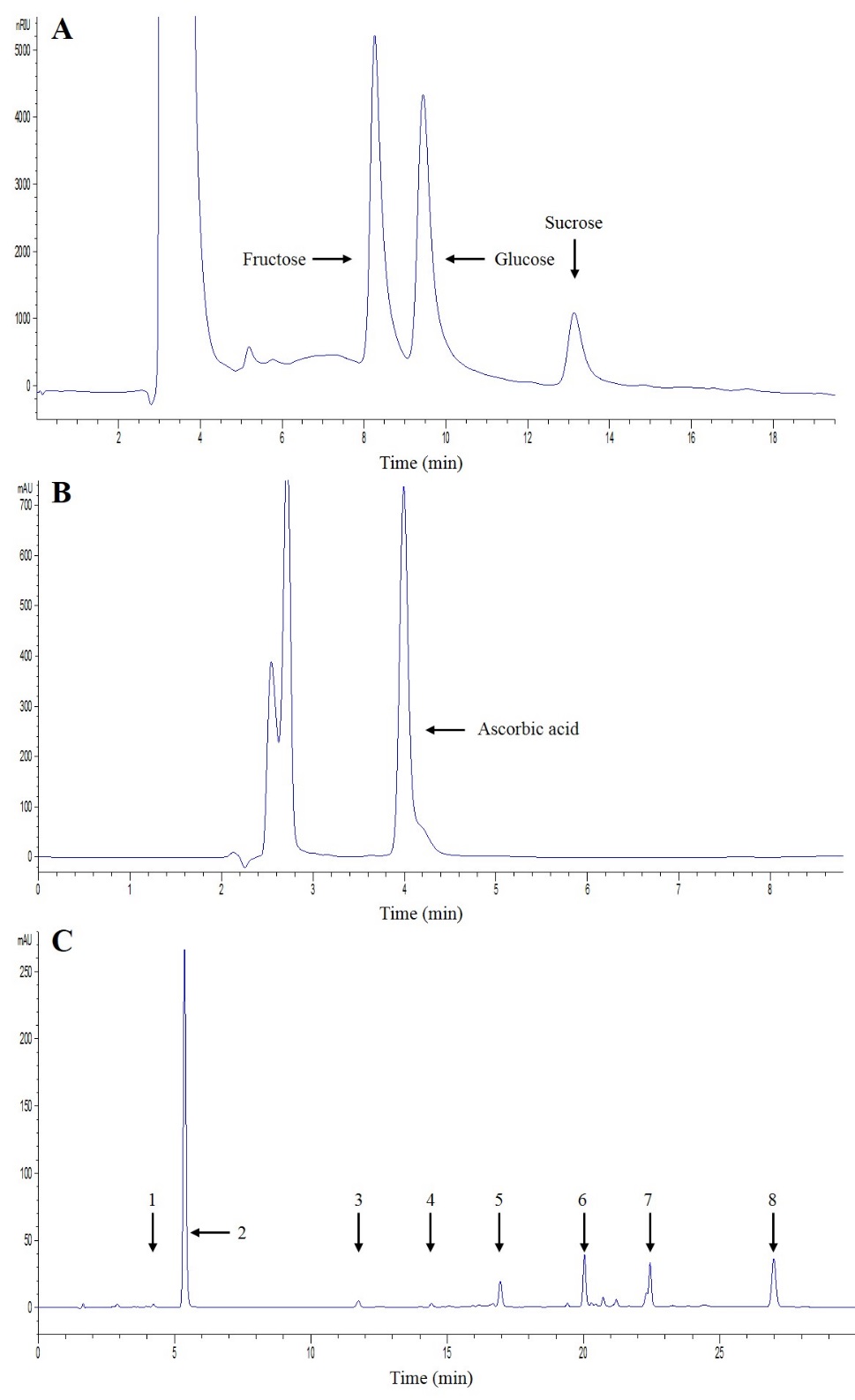

Supplement: Supplementary Figure 1 — Appearance of different baby mustard lateral buds with bags during storage at 4°C under different MAP treatments. M0 indicates packaging with no holes; M1 indicates packaging with 6 mm in diameter holes; M2 indicates packaging with 12 mm in diameter holes. [file Data_Sheet_1.docx]
